# The fine-tuning of NPQ in diatoms relies on the regulation of both xanthophyll cycle enzymes

**DOI:** 10.1038/s41598-021-91483-x

**Published:** 2021-06-17

**Authors:** Lander Blommaert, Lamia Chafai, Benjamin Bailleul

**Affiliations:** 1 Laboratory of Chloroplast Biology and Light Sensing in Microalgae, UMR 7141, Centre National de La Recherche Scientifique (CNRS), Sorbonne Université, Institut de Biologie Physico-Chimique, 75005 Paris, France; 2 Present Address: Department of Estuarine and Delta System, NIOZ Royal Netherlands Institute for Sea Research, PO Box 140, 4400 AC Yerseke, The Netherlands

**Keywords:** Biochemistry, Biophysics, Physiology, Plant sciences

## Abstract

Diatoms possess an efficient mechanism to dissipate photons as heat in conditions of excess light, which is visualized as the Non-Photochemical Quenching of chlorophyll *a* fluorescence (NPQ). In most diatom species, NPQ is proportional to the concentration of the xanthophyll cycle pigment diatoxanthin formed from diadinoxanthin by the diadinoxanthin de-epoxidase enzyme. The reverse reaction is performed by the diatoxanthin epoxidase. Despite the xanthophyll cycle’s central role in photoprotection, its regulation is not yet well understood. The proportionality between diatoxanthin and NPQ allowed us to calculate the activity of both xanthophyll cycle enzymes in the model diatom *Phaeodactylum tricornutum* from NPQ kinetics. From there, we explored the light-dependency of the activity of both enzymes. Our results demonstrate that a tight regulation of both enzymes is key to fine-tune NPQ: (i) the rate constant of diadinoxanthin de-epoxidation is low under a light-limiting regime but increases as photosynthesis saturates, probably due to the thylakoidal proton gradient ΔpH (ii) the rate constant of diatoxanthin epoxidation exhibits an optimum under low light and decreases in the dark due to an insufficiency of the co-factor NADPH as well as in higher light through an as yet unresolved inhibition mechanism, that is unlikely to be related to the ΔpH. We observed that the suppression of NPQ by an uncoupler was due to an accelerated diatoxanthin epoxidation enzyme rather than to the usually hypothesized inhibition of the diadinoxanthin de-epoxidation enzyme.

## Introduction

Most photosynthetic organisms face the same problem in strong light conditions: as the absorption of too much light energy can result in oxidative damage to the photosystem II (PSII) core, they must be capable of dissipating excess energy as heat^1^. This is visualized as the Non-Photochemical Quenching (NPQ) of chlorophyll fluorescence and comprises different components and underlying mechanisms, which were originally resolved in plants based on NPQ relaxation kinetics^2^: transthylakoidal proton gradient (ΔpH)-dependent quenching (qE), state transitions (qT) and photoinhibition (qI).

The main NPQ component in plants is qE, which responds rapidly to changes in light intensity through protonation of the Photosystem II subunit PsbS^3^ by the acidified lumen. A low pH in the lumen also activates the lumenal xanthophyll cycle (XC) enzyme Violaxanthin De-Epoxidase (VDE)^4^, which then dimerizes and de-epoxidizes violaxanthin (Vx) to zeaxanthin (Zx)^5^. While Zx enhances the magnitude of qE, it is not strictly required for it^6^. More recently, a PsbS-independent NPQ component, qZ, has been identified which kinetically follows the Zx concentration and is relevant during steady state (> 10 min) light conditions^7–9^. While Kress and Jahns^9^ argue against a direct involvement of Zx as the quencher itself in qZ, they report a strong correlation between qZ and Zx in plants lacking PsbS as well as in WT plants, when the rapidly relaxing qE component was excluded. Due to the correlation between qZ and Zx, qZ relaxes slower than qE as Zx needs to be epoxidized back to Vx. This is accomplished by the Zeaxanthin Epoxidase (ZEP) which requires O_2_ and NADPH as co-substrates^10^ and is located at the stromal side of the thylakoid membrane^11^. Lastly, a new quenching term ‘qH’ was coined to distinguish sustained photoprotective quenching from fluorescence quenching due to inactivation of the PSII core protein D1, a process called qI or photoinhibition^12^. State transitions also participate to the light-induced decrease of the chlorophyll fluorescence and are termed ‘qT’, even though qT does not correspond to an enhanced heat dissipation of absorbed light energy^13^.

Diatoms are the main primary producers in well-mixed waters, characterized by strong fluctuations in light intensity^14^, and exhibit a fast-responding NPQ component which is dependent on the xanthophyll cycle. Its underlying mechanism is undoubtedly different from plants. Firstly, diatom genomes lack the PsbS gene^15,16^ but possess LHCX genes (related to LHCSR proteins in green algae) that are strictly required for NPQ^17–21^. Interestingly, diatom LHCX proteins do not possess all the amino-acid residues which are responsible for a switch from light harvesting to energy dissipation mode in green algae upon protonation^22,23^ and miss a part of the C-terminus that is located in the thylakoid lumen^17^. Secondly, diatoms can accumulate Vx-cycle pigments after prolonged strong light conditions^24^, but their main XC comprises only two pigments^24^: diadinoxanthin (Ddx) which is reversibly converted into the de-epoxidized diatoxanthin (Dtx). The *Phaeodactylum tricornutum* genome contains multiple genes that are related to the VDE and ZEP genes in plants^25^. One gene ‘VDE’, also named the Diadinoxanthin De-Epoxidation (DDE) enzyme is closely related to the VDE in plants and is probably the main Ddx (and Vx) de-epoxidation enzyme in *P. tricornutum*^26, 27^. Also two ‘VDE-like’ (VDL) and two ‘VDE-related’ (VDR) enzymes are present from which the VDL1 and VDL2, probably do not contribute to the xanthophyll cycle, but are rather involved in de novo pigment synthesis^27^. Three different ZEP genes (ZEP1-3) are found in *P. tricornutum*^25^ which seem to be differentially expressed^28,29^, but their relative activity in diatoxanthin epoxidation are not known to date^30,31^. Finally, in contrast to Zx in plants, Dtx is mandatory for NPQ generation in diatoms^32^.

In all pennate diatoms studied so far, NPQ is proportional to the amount of Dtx^33–39^. Despite reports suggesting a loss of this correlation at the very onset of low light illumination^26,40^, this linear relationship between NPQ and Dtx is very robust in the model diatom *P. tricornutum*. It has been confirmed during steady-state illumination, for cells grown in very different conditions^19, 33,34,37,38^ and remains true during the relaxation in any light intensity and regardless of the presence of uncouplers^41^. The factor of proportionality between NPQ and Dtx remains the same as long as the concentration of NPQ actors (LHCX proteins, total xanthophyll pool) does not change and is modified only as long-term high light acclimation processes take place (expression of LHCX isoforms, de novo synthesis of xanthophylls). The flexible NPQ proportional to Dtx in diatoms should therefore be regarded as a type of (fast) qZ. The simple relationship between NPQ and Dtx is expected if Dtx *itself* is a homogenous Stern–Volmer quencher^42^, however, other more complex NPQ models have been proposed, involving two different quenching sites^43,44^. In some centric diatom species, the relationship between Dtx and NPQ is not linear ^37,45^, which suggests that a qE component, similar to the one in plants, is superimposed to the qZ-type component. The existence of two quenching sites could explain this complex behavior in some centric diatoms, but is difficult to reconcile with the strong linearity between NPQ and Dtx in pennates^46^. Disregarding the conceptual NPQ model and the nature of the quencher, the (short-term) NPQ kinetics in *P. tricornutum* are only determined by the operation of xanthophyll cycle enzymes.

The regulation of the activity of the VDE enzyme has been studied in vitro, demonstrating its pH-dependency^27,47,48^. In addition to pH-regulation, VDE/DDE enzymes are sensitive to the concentration of ascorbate^47^ and could be redox regulated as they contain a number of conserved cysteine-residues^49,50^. This feature has been exploited to inhibit the VDE enzyme in diatoms by the reducing agent dithiothreitol (DTT), allowing to study the regulation of the ZEP enzymes^41,51,52^. The regulation of ZEP in diatoms is particularly relevant, since due to the linear Dtx/NPQ relationship^33–35^, Dtx molecules need to be epoxidized back to Ddx to switch the antenna system from an energy dissipating to a light harvesting mode^41^. Studies using DTT indicate that the Dtx epoxidation rate in diatoms is slowed-down under saturating light in *P. tricornutum*^41^, but the overall light-dependency of the ZEP is not known. Further insight into the regulation of XC enzymes has been obtained using uncouplers, which dissipate the proton motive force across the thylakoid membrane, including the ΔpH component. Based on the suppression of NPQ by uncouplers^53,54^, it was proposed early on that the VDE enzyme is activated in vivo during high light conditions by a drop in lumenal pH. This conclusion remains questionable as ZEP activity is accelerated by the addition of uncouplers under saturating light conditions^41^, which could be sufficient to explain the inhibition of NPQ by uncouplers. Two observations in diatoms suggest that VDE is already active in the dark and is only slightly faster in high light: (1) the pH-optimum of diatom VDE is shifted to higher, almost neutral, pH values in vitro, compared to the plant VDE^47,48^ and could be due to differences in the C-terminal domain containing more charged residues in plants^25^, and (2) a considerable proton-motive-force is present in the dark in diatoms^55^. A VDE that is continuously active, regardless of the lumenal pH changes, is therefore a possibility in diatoms, and would explain why Ddx de-epoxidation and concomitant NPQ development can be observed in dark conditions^51,53^. Recent observations in the pinguiophyte (sister-group to diatoms) *Phaeomonas sp.* indicate that NPQ is primarily regulated by the ZEP enzyme in this species, by effectively avoiding Zx accumulation and hence NPQ development in low light conditions, but not in oversaturating conditions and in darkness^56^. This highlights the fact that, since both enzymes can be active at the same time^29^, NPQ regulation should represent the interplay between both enzymes. However, the role of the epoxidation enzymes has to our knowledge not explicitly been included in any diatom NPQ regulation model, although it is taken into account in NPQ models for plants.

To answer these pending questions regarding XC regulation in diatoms, we wanted to assess the light dependency of the epoxidation as well as de-epoxidation reactions in the model diatom *P. tricornutum *in vivo, without using pharmacological treatments. In this work, the ‘ZEP’ and ‘VDE’ activity corresponds, respectively, to the activity of all three putative ZEPs encoded in the genome and the combined activity of VDE plus other enzymes potentially involved in diadinoxanthin de-epoxidation (VDL, VDR). We exploited two characteristics of *P. tricornutum*: (1) the one-step epoxidation/de-epoxidation reaction makes it a very easy kinetic system to model, and (2) the linearity between NPQ and the de-epoxidized xanthophyll Dtx enables to probe the activity of the XC with a non-invasive tool: fluorimetry. In addition, we used this approach to investigate whether and how the two enzymatic reactions of the XC acclimate to the light environment to ensure an appropriate light dependency of NPQ. Moreover, in our hands, the suppression of NPQ by uncouplers is due to changes in the rate of ZEP rather than that of VDE, contrary to what is usually assumed in the literature.

## Materials and methods

### Culture conditions

Wild-type *Phaeodactylum tricornutum* (Bohlin) Pt1 8.6 (CCMP 2561) was grown in Multitron pro incubators (Infors HT, Bottmingen, Switzerland) at 19 °C in Erlenmeyers, containing 50 ml autoclaved Provasoli’s enriched f/2 seawater medium using Instant Ocean artificial sea salt (33 g L^-1^) (Instant Ocean Spectrum Brands, USA). In semi-chemostat mode, cells were grown in a continuous light regime of 70 μmol photons m^−2^ s^−1^ (white LED panel, JbeamBio, La Rochelle, France) with agitation (100 rpm), whereby one third of the culture was replaced daily by fresh medium. In such a treatment, cell density remained about 1 10^7^ cells ml^−1^ and were determined using a Multisizer 3 Coulter counter (Beckman Coulter, Brea, USA). The third of culture volume removed from the Erlenmeyer was used for measurements. In addition, *P. tricornutum* was grown in batch mode, without agitation, and acclimated for at least two weeks to low light (LL, 13 μmol photons m^−2^ s^−1^), medium light (ML, 70 μmol photons m^−2^ s^−1^) and high light (HL, 280 μmol photons m^−2^ s^−1^). Cells were kept in early exponential phase by diluting every 3–4 days and were harvested at a concentration between 1 and 2 10^6^ cells mL^−1^.

### Chlorophyll *a* fluorescence

Cells were sampled in growth conditions, in the second half of the light period for batch cultures and directly pipetted into 1 ml cuvettes. Measurements were conducted at ambient temperature and started not more than 1 min after sampling. Fluorescence measurements were conducted with a home-made pump-probe fluorometer^57^. We spaced saturating pulses (250 ms, 4210 µmol photons m^−2^ s^−1^) at least one minute apart because we verified that a higher frequency of saturating pulses could generate photoinhibition. The photosynthetic efficiency of PSII (ΦII) was calculated as (F_m_’–F’)/F_m_’ with F’ the fluorescence in illuminated cells and F_m_’ the corresponding maximal fluorescence (following the saturating pulse) in illuminated cells. The relative electron transport rate (rETR) was calculated as ΦII.I. The absorption cross-section of the PSII antenna (σPSII) was calculated in LL and HL cultures as described by Tian et al.^58^ on five times concentrated cells. The area above the fluorescence induction curve in presence of DCMU, with the extrapolated F_0_ (see Tian et al.^58^ for details) and F_m_ normalized to 0 and 1, respectively, corresponds to the average time for a stable closure of a PSII reaction center. NPQ was calculated as (F_m_–F_m_’)/F_m_’, where F_m_ is the reference maximal fluorescence of the low light adapted cells. Like Berne et al.^56^ we used the maximal fluorescence measured under 18 µmol photons m^−2^ s^−1^ (hereafter referred to as reference light) as the reference because that was the condition which maximized F_m_’ and minimized the Dtx content. To distinguish the rapidly relaxing component of NPQ, here termed ‘qZ’, from qI we calculated qZ as (F_m_’’–F_m_’)/F_m_’, with F_m_’’ being the maximal fluorescence after qZ relaxation in reference (18 µmol photons m^−2^ s^−1^) light conditions.

Two modes of fluorescence/NPQ measurements were used. For modelling the XC kinetics of cells in semi-chemostat mode, we avoided perturbation of the cells and therefore did not resuspend the cells during the experiment. The fluorescence levels were therefore slightly biased by sedimentation over the 20 to 40 min experiments. To correct for sedimentation, the same cells were kept for 45 min at 18 µmol photons m^−2^ s^−1^ (the reference light) and F_m_’ values were measured every minute and the measured (small) slope was used to correct the fluorescence kinetics curves for sedimentation. In the second mode of measurements, we used dithiothreitiol (DTT, Sigma-Aldrich, Munich, Germany) to inhibit the de-epoxidation reaction. We illuminated cells with a saturating actinic light and once a steady state NPQ of ~ 1–1.5 was reached, the actinic light was set to the value of interest, just after the last saturating pulse. DTT (200 µM, from a 100 mM stock in milliQ water) was added 10 s before this last saturating pulse, a time which is sufficient for the DTT to efficiently inhibit the de-epoxidase (data not shown). As the addition of DTT induced a resuspension of the cells, we eliminated the potential bias by resuspending the cells by pipetting up and down, after each saturating pulse throughout the experiment. The resuspension slightly decreased the average light irradiance experienced by the cells in the optical path.

NH_4_Cl was used at a final concentration of 5 mM (Sigma-Aldrich, Munich, Germany) from a 500 mM stock solution in milliQ water.

### Pigment analyses

Pigments of cells in semi-chemostat mode were measured by sampling inside the cuvette of the fluorometer during steady-state NPQ conditions and during relaxation in darkness. To ensure a direct comparison between pigments and NPQ, cells were resuspended 10 s before the saturating pulse and sampled (50 µL) directly after, in front of the detector. The sample was immediately added to 950 µl methanol (100%), briefly vortexed and snap frozen in liquid nitrogen not more than 10 s after sampling, and then stored at − 80 °C before analysis. Pigments were analyzed according to Berne et al.^56^, using a Shimadzu Prominence-I LC-2030C 3D HPLC (Shimadzu Corporation, Kyoto, Japan) equipped with a Waters Nova-Pak C18 4 µm 3.9 × 150 mm column (Waters corporation, Milford, USA).

### Statistics and curve fitting

Relative ETR (rETR)-photosynthetic light intensity curves were fitted by rETR = rETR_m_.(1-exp(−α.I/rETR_m_)), whereby α is the initial slope, rETR_m_ the maximum rETR value. E_k_, the light saturation parameter, is calculated as rETR_m_/α^59^. NPQ-photosynthetic light intensity curves were fitted by NPQ = NPQ_m_.I^n^/(E50_NPQ_^n^ + I^n^), whereby E50_NPQ_ is the light intensity for which 50% of the maximum NPQ value (NPQ_m_) is reached and n represents the sigmoidicity parameter of the function^60^. The NPQ vs I curve shown in Fig. 2a was obtained while excluding the point in the dark. The epoxidation rate constant (ke) vs light intensity curves where fitted with the function ke(I) = (A_1_ + A_2_.(1−exp(−x/I_1_)).exp(−x/I_2_) + A_3_, with the constant I_1_ representing the light saturation parameter for epoxidation and I_2_, the inhibition rate constant of epoxidation under high light regime, A_1_ is the epoxidation rate constant in the dark, A_2_ is the light-dependent epoxidation activation and A_3_ is the epoxidation rate constant limit under high light. For fitting the light dependency of rETR, NPQ and enzyme rate constants, data points from individual light-curve experiments were compiled before fitting as some individual experiments contained only a few points. Curve-fitting was conducted using Origin 2018 software (OriginLab, Northampton, USA).

For fits of the NPQ relaxation and calculation of the rate constants of ZEP (ke) and VDE (kd) at a given light intensity, we fitted the experimental data with the function: NPQ(t) = y0 + A.exp(−t/(t + t0).k.t). The term t/(t + t0) was introduced to take the short lag (typically tens of seconds) into account which was observed in NPQ relaxation upon a shift in light intensity. t0 was constrained to values between 0 and 1, so that the lag only significantly changed the first tens of seconds during relaxation (see Supplementary Fig. S2). The values of y0 and k were used to estimate the VDE and ZEP rate constants (see results section).

Statistical analyses were conducted with R: A Language and Environment for Statistical Computing (R foundation for statistical computing, Vienna, Austria). Significant differences between two groups (DTT vs absence of DTT per light intensity, or ke and kd between different light intensities in semi-chemostat cultures) were evaluated by unpaired Student’s t-tests, whereas differences between parameters obtained using different pre-illumination intensities were compared using paired Student’s t-tests. Normality was tested with the Shapiro Wilk test, homogeneity of variances with the Levene’s test. The Two sample Wilcoxon was used as a non-parametric alternative for the student’s t-test when required. Significant differences between individual curves (rETR, NPQ and ke) were identified by comparing a reduced model (considering data from different acclimation conditions as one dataset and fitting hence one set of parameters), to a full model (fitting different acclimation conditions with a separate set of parameters) using the anova_nlslist statement in the R-package NLShelper. As a reasonable estimate for I_1_ in the full model was not obtained for the individual curves, and is not the primary goal of this work, I_1_ was set to the value of the reduced model estimate.

## Results

### Using a modeling approach to resolve the epoxidation and de-epoxidation rate constants

The main aim of this paper was to estimate the in vivo activity of both enzymes of the xanthophyll cycle, the VDE and the ZEP by using the peculiarities of the NPQ of *Phaeodactylum tricornutum*. *P. tricornutum* was grown in semi-chemostat mode in order to keep a constant physiology and cell concentration for a long period of time (see Materials and Methods). The culture reached a steady-state condition in which the total XC pool (Ddx + Dtx) was 27 ± 1 mol (100 mol Chl *a*)^-1^. Vx-cycle pigments were not detected in these culture conditions. Most importantly, NPQ was linearly correlated with Dtx in steady-state NPQ conditions, as well as during relaxation which is a requirement for the treatment below (Supplementary Fig. S1). Because there was Dtx present in steady-state at the light intensity for which F_m_’ was maximal, we obtained Eq. (1) with [Dtx] representing the de-epoxidation state [DES] of the XC pool, rather than the absolute concentration:

1 $$NPQ=2.49 [Dtx]-0.20$$

In these culture conditions, NPQ reached a high maximal value (NPQ_m_) of 1.50 ± 0.02, and its relaxation was relatively slow, allowing to follow the kinetics with a good temporal resolution. The other photosynthetic parameters are listed in Table 1. On top of the linear relationship between NPQ and Dtx, two other requirements needed to be fulfilled by our experiments to model the XC in vivo using fluorescence. The second requirement was the absence of photoinhibition. Indeed, if NPQ contained photoinhibition (qI) on top of the Dtx-dependent qZ/qE component, the linear relationship between NPQ and Dtx would have been lost. The third requirement relates to the behavior of the XC enzymatic reactions: the enzymatic reactions needed to be first order reactions, with the rate constants responding rapidly to a change of light intensity and remaining constant afterwards. Then, the rate of an enzymatic reaction (epoxidation, or de-epoxidation) equals the enzymatic rate constant (ke or kd, respectively) times the concentration of its substrate ([Ddx] or [Dtx], respectively):

$$vd=kd.\left[Ddx\right]\;and\;ve=ke.\left[Dtx\right]$$

**Table 1:** Photophysiological parameters of *P. tricornutum* acclimated to LL, ML, HL and semi-chemostat conditions.

	LL	ML	HL	Semi-chemostat
F_v_/F_m_	0.63 ± 0.03	0.69 ± 0.02	0.52 ± 0.05	0.61 ± 0.01
α	0.65 ± 0.02	0.68 ± 0.02	0.56 ± 0.01	0.67 ± 0.01
E_k_ [μmol photons m^−2^ s^−1^]	105 ± 10	209 ± 16	389 ± 27	105 ± 6
rETR_m_[μmol electrons m^−2^ s^−1^]	68.6 ± 2.0	142.5 ± 3.8	217.7 ± 5.5	67.3 ± 1.3
NPQ_m_	1.33 ± 0.03	2.05 ± 0.06	4.00 ± 0.48	1.50 ± 0.02
NPQ_E50_ [μmol photons m^−2^ s^−1^]	231 ± 8	452 ± 12	995 ± 112	159 ± 3
NPQ_n_	3.40 ± 0.33	3.78 ± 0.35	2.62 ± 0.41	4.30 ± 0.26
I_2 epox_ [μmol photons m^−2^ s^−1^]	101 ± 15	249 ± 31	358 ± 76	96 ± 43
(Dtx + DDx)/100 Chl *a* [mol mol^−1^]	5.73 ± 1.31	8.09 ± 0.92	23.36 ± 1.87	26.74 ± 0.99
NPQ_E50_/E_k_	2.20 ± 0.28	2.16 ± 0.22	2.56 ± 0.47	1.51 ± 0.12
I_2 epox_/E_k_	0.96 ± 0.23	1.19 ± 0.24	0.92 ± 0.26	0.91 ± 0.46

F_v_/F_m_ and (Dtx + Ddx)/100 Chl *a* values ± standard deviation. All other parameters represent fitted values and are given ± standard error obtained from fitting with Origin software.

Provided that [Ddx] and [Dtx] represent the epoxidation and de-epoxidation states of the XC pool, respectively, rather than absolute concentrations, then [Ddx] + [Dtx] = 1 and changes in the Dtx concentration per unit of time $(\frac{d\left[Dtx\right]}{dt}$) thus equal the amount of Dtx produced by de-epoxidation minus the amount removed by epoxidation:

2 $$\frac{d\left[Dtx\right]}{dt}=-\frac{d\left[Ddx\right]}{dt}= vd-ve= kd.\left[Ddx\right]-ke.\left[Dtx\right]=kd.\left(1 -\left[Dtx\right]\right)- ke.[Dtx]$$

The kinetics of changes from a light intensity (called Ip for preceding intensity) to another (I, relaxation intensity) can be obtained by integrating the differential Eq. (2):

3 $$\left[Dtx\right]\left(t\right)=\left(\frac{kd(Ip)}{kd(Ip)+ke(Ip)}-\frac{kd(I)}{kd(I)+ke(I)}\right)\mathrm{exp }\left(-\left(kd\left(I\right)+ke\left(I\right)\right).t\right)+\frac{kd(I)}{kd(I)+ke(I)}$$

where $\frac{kd(Ip)}{kd(Ip)+ke(Ip)}$ and $\frac{kd(I)}{kd(I)+ke(I)}$ represent the steady-state DES at preceding light Ip and relaxation light I, respectively. Using the linear relationship between NPQ and [Dtx], the kinetics of NPQ evolution can be obtained by combining Eqs. (1) and (3):

4 $$\mathrm{NPQ}\left(t\right)=2.49. \left(\frac{kd(Ip)}{kd(Ip)+ke(Ip)}-\frac{kd(I)}{kd(I)+ke(I)}\right) .\mathrm{exp}\left(-\left(kd\left(I\right)+ke\left(I\right)\right).t\right)+\frac{kd(I)}{kd(I)+ke(I)}-0.20$$

**Figure 1:**
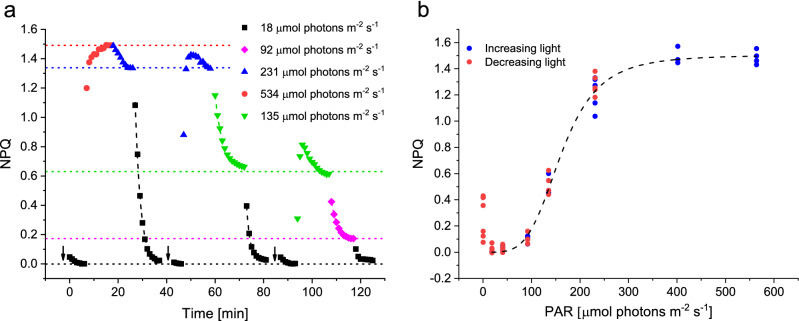
Representative subset of the experiments that were used to feed the XC model (panel **a**). Different symbol and line colors represent different light intensities at which samples were exposed until a steady-state was reached (indicated by dotted lines). Light-induced changes in NPQ were fitted with mono-exponential decay functions, represented by dashed lines. A new sample (indicated by an arrow) was used for each experiment. NPQ levels above zero right after changing a sample indicate the presence of small but significant NPQ in growth conditions which relaxed in reference conditions (black squares). Panel (**b**) shows the superposition of the measured NPQ values after NPQ induction (increasing light, blue dots) and relaxation (decreasing light, red dots). The averages per light intensity of each experimental day are plotted.

Equation (4) thus describes the exponential decay of NPQ from a given light intensity to a lower one with only two unknowns, ke and kd. After fitting experimental data with this function, the rate of the exponential decay represents *the sum* of kd and ke, whereas the asymptote (the steady-state final NPQ value) represents kd/(kd + ke). Solving this system of two equations with two unknowns gives estimates for the rate constants of ZEP as well as VDE, simultaneously and without using pharmacological treatments (which often come with secondary effects). Figure 1a shows a representative subset of the experiments we conducted to feed the model and test our assumptions. In brief, diatom samples were first adapted to 18 μmol photons m^-2^ s^-1^ to obtain the highest possible F_m_’, used as the reference to calculate NPQ (see Materials and Methods). Then we exposed the samples to sequential light intensity shifts to modulate NPQ. To avoid the development of photoinhibition (qI) and *de-novo* synthesis of Dtx, we used a new sample regularly, avoided using multiple sequential high light intensities and verified the absence of qI, evidenced by a complete NPQ relaxation in 18 μmol photons m^-2^ s^-1^ and equal steady-state NPQ levels regardless of the light history of the samples (dotted lines in Fig. 1a). The absence of qI is confirmed by the superposition of the NPQ values after NPQ induction (increasing light) and relaxation (decreasing light) (Fig. 1b).

During increasing light conditions, NPQ often showed a transient peak before relaxing again to a steady-state value, indicating that the time needed by enzymatic rate constants to adjust to their new light condition and reach a steady-state was not short enough, compared to the NPQ induction (Fig. 1a). This was particularly evident for the NPQ generation under intermediate light intensities (Fig. 1a, for instance at 231 μmol photons m^-2^ s^-1^ (blue triangles) and 135 μmol photons m^-2^ s^-1^ (green inverted triangles)). As this is a violation of our third assumption to model NPQ kinetics, we did not include data involving increasing light intensities. The situation was different during decreasing light intensities, whereby after a lag of generally few tens of seconds (see Supplementary Fig. S2 and Discussion), NPQ relaxation followed a clear mono-exponential decay function (dashed lines in Fig. 1a, see also Fig. 6 and Supplementary Fig. S4). This indicates that the reactions were indeed order 1 reactions and that XC enzymes reached their steady-state rate constants relatively fast after a shift from a higher to a lower light intensity. We took into account the fact that a lag was observed at the light shift before a mono-exponential decay of NPQ was evident (see Material & Methods). We also verified that the intensity of the preceding light influenced the lag but had only minor effect on the values of kd and ke, compared to biological variations or the effect of the relaxation light (see Supplementary Fig. S3 and Fig. S4 for the influence of the preceding light intensity and duration respectively). Only for NPQ relaxation in darkness, the preceding light intensity strongly impacted the kd and ke estimates (see Supplementary Fig. S3), resulting in a large dispersion of kd and ke values depending on the light protocol used (see “Discussion”).

**Figure 2:**
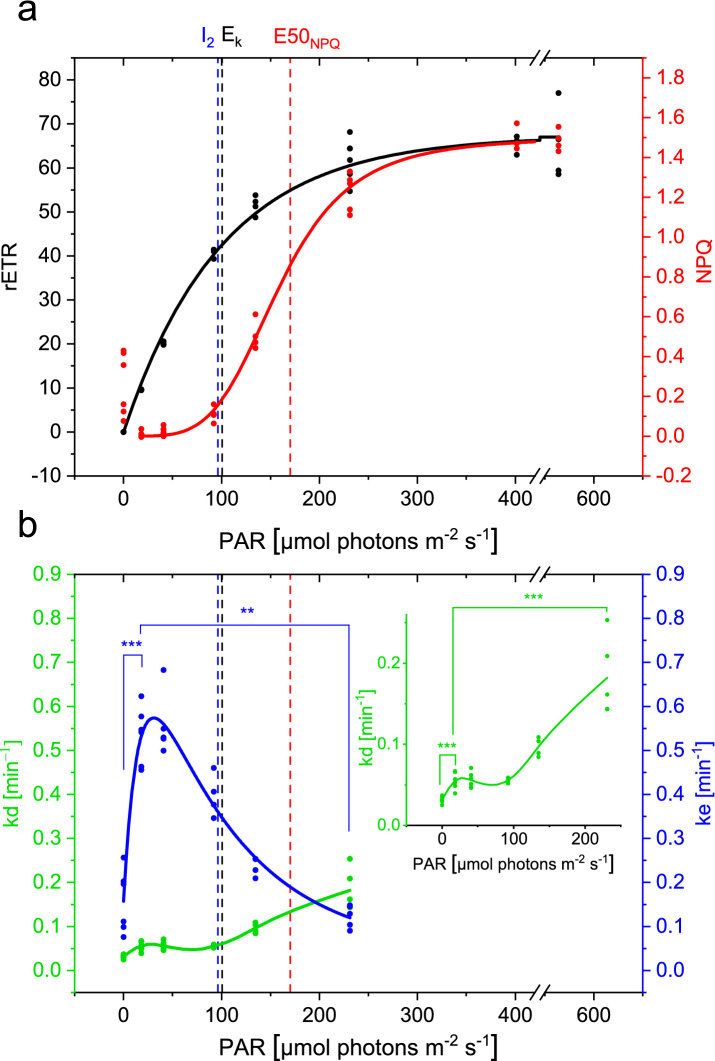
Steady-state rETR (black) and NPQ (red) values (panel **a**) and kinetic rate constants of epoxidation (ke, blue) and de-epoxidation (kd, green) (panel **b**) as a function of the green light intensity. Ke and kd were obtained from the NPQ relaxation kinetics and steady-state NPQ levels, as shown in Fig. 1. NPQ, rETR and ke curves were fitted by functions described in Materials and Methods, while kd data points were connected by a spline curve. Dashed lines represent the inhibition rate of ke (I_2_, blue), the light-saturation parameter (E_k_, black) and the light intensity whereby 50% of the maximum NPQ is reached (E50_NPQ_, red). For each light intensity, at least 3 experiments were conducted and all datapoints represent the average value of each experimental day. Significant differences of ke and kd values between reference light and either darkness or 231 μmol photons m^-2^ s^-1^ are indicated with * for P < 0.5; ** for P < 0.01 and *** for P < 0.001 in blue for ke and in green for kd in the inset in the right upper corner.

The three requirements listed before are now met (no qI, first order kinetic reactions and proportionality between Dtx and NPQ), validating our procedure to model the NPQ kinetics. Fitting the NPQ relaxation kinetics and calculation of the steady-state NPQ levels allowed to obtain the rate constants of the de-epoxidation (kd) and epoxidation (ke) enzymatic reactions according to light intensity. The light intensity for which half the maximal value of NPQ was reached (E50_NPQ_) was 159 ± 3 μmol photons m^-2^ s^-1^, being higher than the optimal light intensity (E_k_), which was 100 ± 6 μmol photons m^-2^ s^-1^, based on the rETR vs I curve (Fig. 2a). We observed a ke optimum in the low light intensity range (Fig. 2b), where photosynthesis is light-limited, as evidenced by the rETR vs. I plot (Fig. 2a). Together with the de-epoxidation rate constants in the light-limited range being minimal, the resulting NPQ was minimal in low light. In darkness, ke was significantly lower than in low light, but still active, explaining the small NPQ values obtained in the dark, as already discussed^48,51,61^. As the light intensity increased, we first observed a drastic decline of ke, before a significant increase of kd, demonstrating the activation of the de-epoxidation reaction. Both enzymatic reactions thus regulate NPQ development in concert to dissipate excess light energy in oversaturating light conditions, whilst optimizing light harvesting in light-limiting conditions.

### Validating the light profiles of ZEP with the de-epoxidation enzyme inhibitor DTT

**Figure 3:**
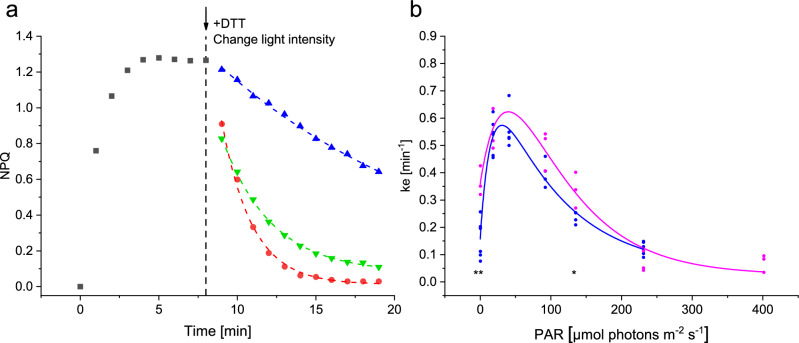
DTT-based method to obtain the epoxidation rate constant ke. (**a**) After reaching a steady-state NPQ (black squares), DTT was added as indicated by a black arrow and a black vertical dashed line, before the light intensity was changed to different light intensities (or kept the same), as represented by green, red and blue symbols (0, 18 and 231 µmol photons m^-2^ s^-1^, respectively). The observed NPQ decay was fitted by a mono-exponential function (dashed colored lines), to obtain the epoxidation rate constant ke. (**b**) The epoxidation rate constant ke in function of light intensity, obtained by using DTT, (magenta) in comparison to the ke-light intensity curve obtained without DTT (in blue), See Figs. 1 and 2b. Three measurements were made for each light intensity. Significant differences are indicated with * for P < 0.5; ** for P < 0.01 and *** for P < 0.001 between ke values obtained using the DTT-based method and the modeling approach in black below the curves and between ke obtained in reference light compared to either dark or 231 µmol photons m^-2^ s^-1^ in magenta above the curves.

To investigate the light dependency of the ZEP rate constant and to compare the output of our method to previous protocols, we performed a second experiment using the same approach as used by Goss et al.^41^. Therefore, we first established a steady-state NPQ level (by illuminating samples for 6 min at 231 µmol photons m^-2^ s^-1^) and followed their NPQ relaxation kinetics after addition of DTT, under different light intensities (See examples in Fig. 3a). We used 200 μM of DTT, which did not affect the overall photosynthetic performance of our samples under low light, but inhibited virtually all qZ under high light (Supplementary Fig. S5). The rationale behind this protocol, previously used in the pinguiophyte *Phaeomonas* sp.^56^, is that when the de-epoxidase is inhibited, the changes in NPQ reflect only the activity of the epoxidase^41^. As illustrated in Fig. 3a, the NPQ decay was the fastest under reference light conditions (red) and slower in the dark (green) or in high light (blue), in agreement with the bell-shaped curve of the ke light dependency (Fig. 2b). The light dependency of ke in the presence of DTT (magenta in Fig. 3b) displayed a similar bell-shaped pattern as in the approach without DTT (blue, from Fig. 2b). The epoxidation rate constant decreased even further in the higher light intensity range which was not accessible with the protocol used in Fig. 2b and reaches zero at 534 µmol photons m^-2^ s^-1^, where no relaxation of NPQ was observed upon addition of DTT. Although the epoxidation rate calculated with and without DTT were very different in darkness (see discussion), and showed a small significant difference also at 92 µmol photons m^-2^ s^-1^, the overall good agreement between the two profiles validates our procedure using no pharmacological treatment (Fig. 3b).

In contrast to what has been proposed earlier, our experiments showed that the epoxidase enzyme was not only inhibited in saturating light conditions^41^, but was already inhibited way before light becomes saturating for photosynthesis. The intensity of half-inhibition of ZEP is surprisingly close to E_k_ suggesting that the inhibition of ZEP is proportional to photosynthetic activity, whereas the activation of the VDE enzyme only started at light intensities above E_k_. Given the very different light range for ZEP inhibition and VDE activation, it seems unlikely that ZEP is regulated by the same mechanism as VDE.

### The effect of light-acclimation during growth on the epoxidation enzyme regulation

**Figure 4:**
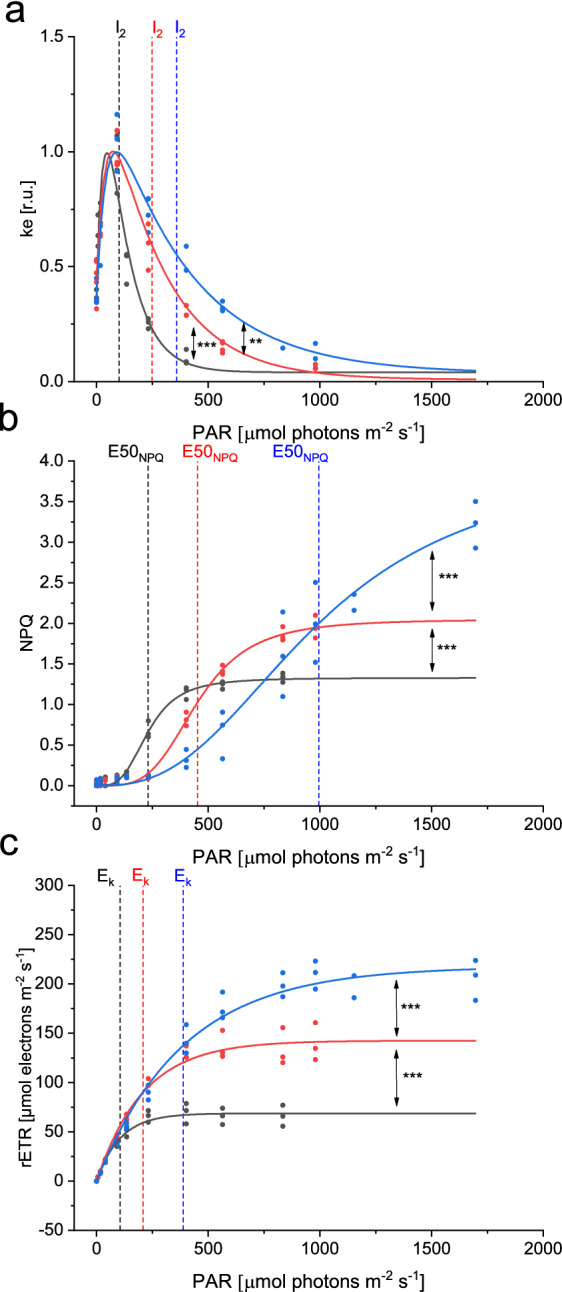
Light dependency of ke (**a**), NPQ (**b**) and rETR (**c**) in cells acclimated to LL (black), ML (red) and HL (blue) conditions. Ke values were obtained using DTT, as shown in Fig. 3a and normalized to their respective maximal value. All individual data points, originating from at least 3 independent cultures are shown. Significant differences between fitted curves are indicated with * for P < 0.5; ** for P < 0.01 and *** for P < 0.001.

To confirm these findings and to test whether the light-dependency of XC enzymes is modified by the light conditions to which the cells acclimate to, we investigated the regulation of the VDE enzyme in cells acclimated to low (LL, 13 µmol photons m^-2^ s^-1^) medium (ML, 70 µmol photons m^-2^ s^-1^) and high light (HL, 280 µmol photons m^-2^ s^-1^) conditions (Fig. 4a–c and Table 1). The maximum NPQ (NPQ_m_) increased and a larger XC-pool was observed as growth light conditions increased (Table 1). We used the DTT-based method to obtain the epoxidation rate constant ke (see discussion and Fig. 3a) with a sufficient concentration of DTT (Supplementary Fig. S6) and exposed samples to 401, 564 and 833 µmol photons m^-2^ s^-1^ for cells grown in LL, ML and HL, respectively, to generate NPQ before the addition of DTT. The resulting light-dependency of ke (Fig. 4a) in all three growth light conditions confirmed the pattern observed in cells grown in semi-chemostat conditions, with an optimum in the light intensities where photosynthesis was light-limited and lower rate constants in darkness and a gradual inhibition with increasing light intensities. Acclimation to increasing growth light intensity significantly altered the light dependency of ke, rETR and NPQ. The half-inhibition intensity (I_2_), the light intensity to reach 50% of the maximum NPQ (E50_NPQ_) and the light saturation parameter E_k_ increased together with the growth light intensity (Fig. 4b, Table 1). Again, the half-inhibition intensity for ZEP was close to E_k_, whereas the half-saturation intensity for NPQ was more than 2 time higher. The shifts in I_2_, E50_NPQ_ and E_k_ could not be attributed to a difference in light absorption, since the PSII absorption cross-section did not change between LL (3.88 ± 0.40 nm^2^) and HL (3.69 ± 0.11 nm^2^) conditions. These results highlight that the light acclimation processes resulting in different rETR and NPQ light-dependency relies at least partly on the regulation of the light dependency of the ZEP rate constants. They also confirm that the epoxidation enzyme is inhibited already at light irradiances lower than the light saturation point (E_k_) and onset of NPQ, in agreement with a different mode of regulation of ZEP and VDE.

**Figure 5:**
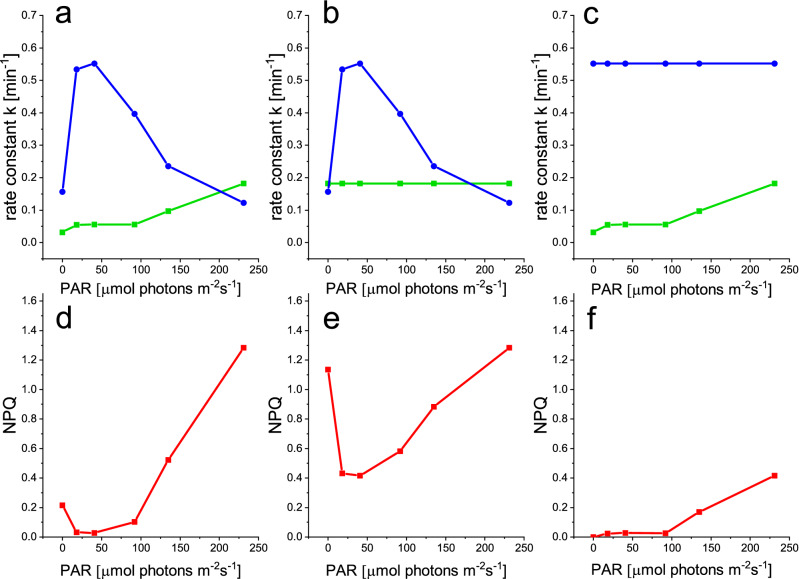
Top panels: three scenarios for regulation of the XC enzymes (blue: ke; green: kd) are shown, with the experimental rate constant values for the two enzymes (**a**) or when only the experimental values for kd (**b**) or ke (**c**) are used, the other one being kept constant. Bottom panels: simulated NPQ values using Eq. (5) for the three scenarios a, b and c are shown in panels (**d**–**f**), respectively.

The results obtained in this work demonstrate that NPQ is not only regulated by the de-epoxidase rate constant but also by the rate constant of the epoxidase enzyme, which can change drastically, depending on light conditions. To highlight the relative roles of the two enzymes better, we simulated what the values of NPQ would be using Eq. (4) for two scenarios of XC enzymes regulation and compared them to the control scenario based on the experimental values obtained in this work (Fig. 5a,d). The first one corresponds to a situation where the de-epoxidase rate constant is not regulated and keeps its maximal experimental value regardless of light intensity (Fig. 5b,e). In that case, the maximal NPQ value would be unchanged compared to the control scenario but there would be NPQ under low light, leading to a useless dissipation of photons in the light-limiting range. The second one, in contrast, postulates that the epoxidase rate constant is independent of light intensity and is equal to its maximal experimental value (Fig. 5c,f). In that case, there would be no quenching under the low light range when light harvesting must be optimized, but the maximal NPQ under saturating light is lower, leading to insufficient dissipation of excess light energy. Altogether, we can conclude that only the combination of the activation of the de-epoxidase and the inhibition of the epoxidase under high light allows to (1) maximize light absorption when photosynthesis is light limited and (2) dissipate efficiently excess light when light absorption exceeds the photosynthetic capacity.

**Figure 6:**
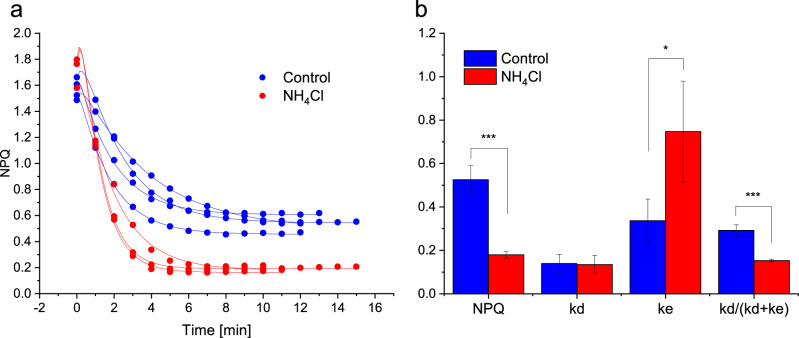
The effect of the uncoupler ammonium chloride (NH_4_Cl) on the relaxation of NPQ (panel **a**) and the steady-state NPQ, obtained kd and ke rate constants and kd/(kd + ke) ratio (panel **b**). Blue curves and bars (control) compared to red curves and bars (5 mM ammonium chloride). All data originates from at least 3 independent cultures and significant differences between ammonium chloride and control parameters are indicated with * for P < 0.5; ** for P < 0.01 and *** for P < 0.001.

Finally, we wanted to use our method to investigate the effect of an uncoupler on NPQ. We chose a protocol whereby the light intensity was decreased from a light intensity for which NPQ was maximal, to a relaxation light intensity just below E50_NPQ,_ for which the steady-state NPQ was ~ 0.5. The addition of 5 mM ammonium chloride significantly decreased NPQ, in agreement with previous reports^41,54^ (Fig. 6a), which indicate that the ratio kd/(kd + ke) decreased with the uncoupler. This inhibition of NPQ by uncouplers was often used as evidence for the regulation of diatom VDE by the lumenal pH in vivo. Our analysis not only confirms previous findings that ZEP is accelerated under high light in the presence of an uncoupler^41^, but it also surprisingly indicates that the rate constant of VDE is not significantly modified by the treatment (Fig. 6b).

## Discussion

Diatoms encounter fast fluctuations in light intensity in their natural habitat^62^ and have therefore developed the ability to rapidly adjust the light utilization of PSII by Non-Photochemical Quenching^32,46^. The regulation of NPQ according to light intensity is directly reflected by the light-regulation of the XC. The family of LHCX proteins is involved in NPQ as well^17,46^, but the robust linear relationship between NPQ and Dtx^33,34^ suggests that they are not the effectors of NPQ, i.e. are not involved in the regulatory mechanism which switches the antenna from a light-harvesting to a heat-dissipation mode. In this work, we fitted a model to the XC kinetics of the diatom *Phaeodactylum tricornutum*. This allowed us to investigate the light-regulation of VDE and ZEP simultaneously in steady-state conditions for the first time, and without the use of pharmacological treatments. As such we demonstrated that the de-epoxidation and the epoxidation enzyme(s) both display a tight light-regulation, though both are regulated by different mechanisms. The light-regulation consists of a high light activation of the VDE and a bell-shaped light-dependency of the ZEP (Fig. 2b). Simulations were performed, where only one of the two enzymes was regulated as experimentally observed, while the other enzyme was not regulated and thus active at its maximum (observed) rate constant (Fig. 5). Those simulations demonstrated that the tight regulation of both enzymes is necessary for NPQ fine-tuning and for providing the optimal compromise between light-harvesting and dissipation of excess energy at all light irradiances. Indeed, when the epoxidase was not regulated, i.e. exhibiting its maximum rate constant at all light intensities, the NPQ was insufficient at saturating light intensities, as proposed earlier^41^. On the contrary, a constant VDE activity would lead to dissipation of photons in low light conditions; with a notch in the NPQ/Dtx curve at the ZEP light-optimum. Interestingly, this situation was observed in the pinguiophyte *Phaeomonas*, where ZEP is the prime XC regulator and VDE seems to have a rather limited regulatory role^56^.

Acclimation to increasing light intensities during growth, resulted in a shift in the light saturation point E_k_ and in the light intensity for which 50% of NPQ was reached (E50_NPQ_), as is generally observed in diatoms^60,63^ (Fig. 4). The maximum NPQ amplitude (NPQ_m_) increased, probably due to the observed higher amount of xanthophyll cycle pigment pool (Ddx + Dtx)^64^ and maybe LHCX content^19^ (Table 1). Interestingly, we observed that the epoxidation reaction inhibition point I_2_ also shifted to higher values and remained very close to E_k_ for the 3 light acclimations, like for the semi-chemostat culture (Table 1). We confirmed that this shift in inhibition was not due to a change in PSII antenna size, which would compensate higher light intensity by an inversely lower PSII absorption cross-section. The regulation of the epoxidation enzyme actually accommodates to changes in the light response of photosynthetic activity. The rather strict relationship between I_2_, E50_NPQ_ and E_k_ observed in this study (and by Serôdio and Lavaud^60^ for E50_NPQ_ and E_k_), implies a strong regulation of the xanthophyll cycle enzymes according to the perceived light intensity. While these results obtained using DTT are valid qualitatively, there are sources of error which make the quantitative interpretation of these results questionable. Errors may be due to technical differences in the way the two experiments were handled: in DTT experiments, we resuspended after each pulse to keep the conditions similar throughout the experiment, hereby mixing highly quenched cells with less quenched cells (due the a heterogenous light climate in the measuring cuvette). This may have shifted the ke vs light intensity curve slightly towards higher light intensities (see Fig. 3b). More important is the significantly higher values of ke in darkness when using DTT (with a high variability in the results, depending on the replicate) and could be due to secondary effects of DTT on the photosynthetic electron transfer chain (Fig. 3b). Despite our attempt to use the lowest concentration allowing VDE inhibition, the rather high concentration of this reducing agent could modify the redox state of several actors of the photosynthetic chain. A reduction of the oxidized form of nicotinamide adenine dinucleotide phosphate NADP^+^ into NADPH, which is the substrate of the epoxidase, could explain the higher rate of the ZEP in the presence of DTT.

The relaxation of NPQ in darkness is not only dependent on the presence or absence of DTT, it is also the unique situation in our dataset where the VDE and ZEP activity was clearly influenced by the light history (the preceding light intensity, see Supplementary Fig. S3). When relaxation occurred in the light, the light history of the cells mainly influenced the lag before the mono-exponential decay; ke and kd values were not significantly modified and the lag did not exceed one minute. In the dark, this influence occurred throughout the NPQ relaxation, which could last up to 30 min. Even though it remains very speculative at this stage, one possibility could be that some of the parameters regulating the activity of VDE and ZEP, like the NADPH, ascorbate pools or the ΔpH show an inertia at the light-to-dark shift which serve as a memory of the previous illumination step. Light exposure during NPQ relaxation would otherwise reset those parameters more rapidly to their steady-state values in their respective relaxation light condition. Regardless of the reason why such an effect of light history on NPQ exists in darkness, it should warn us to be extremely careful when handling NPQ experiments, and favor low light relaxation instead of dark adaptation when one wants to set a reference situation in terms of physiology.

The light-dependency of the two XC enzymes reveals some features that can be informative on their regulation mode (redox, substrate, phosphorylation, pH,…). When light energy oversaturates the capacity for photosynthetic electron transport, i.e. beyond the light saturation parameter E_k_, a strong increase in the VDE activity and concomitant Dtx production, resulting in NPQ was indeed observed and probably reflects the ΔpH-activation of VDE^27,47,48^. The pH dependency of the VDE in plants is associated with the pH-dependent dimerization of the enzyme, which is required for its catalytic activity^5^. While the in vivo regulation of the VDE by the lumenal pH has been demonstrated in several species through the use of the K^+^/H^+^ exchanger nigericin^45,65,66^, this could not be done in *P. tricornutum*, because of the ambiguous effect of nigericin, which either does not enter the cells^51,54^ or enters and increases F_m_ in darkness^61^. High concentrations of uncouplers like NH_4_Cl or carbonylcyanide m-chlorophenylhydrazon (CCCP) do inhibit NPQ in this diatom^54,61,67,68^, but whether this is due to the suppression of the ΔpH, to the arrest of the linear electron flow or an increase in ZEP activity^41^ is not clear in the absence of control of the electron transport rate in these conditions. We therefore consider that the regulation of VDE by the lumenal pH remains the most parsimonious explanation for its observed high light activation in our dataset, but we acknowledge that there is no clear demonstration of this yet in *P. tricornutum*. In darkness, NPQ was only slightly higher than in low light conditions. As the VDE enzyme in diatoms has a pH-optimum that has been shifted (in comparison to plants) to almost neutral pH^27,47,48^, only a slight acidification of the thylakoid lumen should activate VDE. The thylakoid lumen could also be acidic in darkness due to the reverse action of the ATP synthase, hydrolyzing ATP produced by the mitochondrion^55^, which was attributed to chlororespiration in earlier reports^51,66,69^. The question of the necessity of a light-activated VDE in *P. tricornutum* was still an open question. Here, we do confirm that a strong activation of the VDE rate constant occurs at a light intensity corresponding to the onset of NPQ (Fig. 2). We did not observe a strong activity of the VDE in the dark, but its activity was sufficient, together with the low activity of ZEP in darkness, to create a small but significant NPQ in darkness.

ZEP in *P. tricornutum* mirrors the NPQ curve and shows a clear optimum in low-light conditions, explaining the highest F_m_ (and the lowest NPQ) in the low light range as well as the fastest NPQ relaxation rate in these conditions. Our experiments in presence or absence of the VDE inhibitor DTT showed that Dtx epoxidation is significantly slower in darkness than at its low-light optimum. In the pinquiophyte *Phaeomonas*, where the epoxidation enzyme is fully inhibited in darkness, the accumulation of significant amounts of the de-epoxidized xanthophyll Zx resulted in an NPQ higher than 2 in the dark^56^. Here, the epoxidation rate constant in the dark could not prevent Dtx accumulation and concomitant NPQ development in prolonged dark acclimation (Supplementary Fig. S7). The acceleration of the ZEP activity under low light compared to darkness has been observed before and was attributed to the light-driven accumulation of the ZEP substrate NADPH^41^.

On top of the high light activation of the VDE, probably mediated by lumenal pH, and the low light activation of ZEP, related to the NADPH availability, ZEP is also inhibited beyond this optimum at higher light irradiance. This light-induced downregulation of the epoxidation reaction allows a net accumulation of Dtx and leads to the onset of NPQ. This third level of regulation, however, is the most difficult to characterize. While the lack of the co-factor NADPH may explain ZEP inhibition in darkness, a similar NADPH deficiency in high light conditions seems unlikely as the NADPH concentration increases with light intensity in *P. tricornutum*^55^, similarly as in *Phaeomonas sp.*^56^. Because of the effect of uncouplers on the activity of ZEP, its regulation by the lumenal pH has been proposed^41^. However, the ZEP enzymes is believed to be located on the stromal side of the thylakoid membrane like in plants^11^, which makes a similar ΔpH regulation as VDE difficult. Here, all our experiments showed the inhibition of ZEP well before the onset of de-epoxidase activation. Not only the ZEP inhibition parameter I_2_ was significantly lower than E_50NPQ_, it was also lower than E_k_: the process of ZEP downregulation was almost saturated at light intensities for which the process of VDE up-regulation did not yet occur. The difference of light-regulation between VDE and ZEP rules out the possibility that the two enzymes share the same regulator. Hence, if VDE is regulated by the build-up of a ΔpH, ZEP seems to be regulated by another factor. It was suggested that a stromal-side localized ZEP could be directly or indirectly sensitive to small pH changes in the stroma^1^, as a pH increase in the stroma could parallel the acidification of the lumen^70^. In this model, the question how a regulation of ZEP by stromal pH could occur at lower light intensity than the regulation of VDE by the lumenal pH, however, remains to be understood. A last possibility, that we favor, is that ZEP is regulated by another mechanism, such as redox regulation, as it is the case for VDE in plants^49^. Plant ZEP may be redox-regulated as well, since it forms intermolecular disulfide bridges which produces oligomers in vitro^71^. Decreasing the amount of M-type thioredoxins or the NADPH-dependent chloroplast thioredoxin system (NTRC) in *A. thaliana*, resulted in a decreased ZEP activity and an increase in Zx in low-light conditions. Together with an increased ΔpH, this resulted in a deregulation of qE^72,73^. A similar redox down-regulation of ZEP in *P. tricornutum* under moderate and high light could explain the light-dependency of the enzyme as the *P. tricornutum* genome encodes a plastid-located M-type thioredoxin and an NTRC protein. The latter however, is putatively located in the periplastidic compartment^74^. Another possible short-term ZEP regulation that has been tested in plants is phosphorylation. Reinhold et al. failed to show an effect on ZEP downregulation of two thylakoid protein kinases^75^ but experiments with the phosphorylation inhibitor alkaline phosphatase, in vitro, indicate a reversible ZEP inhibition by phosphorylation in *Oryza sativa*^76^. Besides the above-mentioned short-term regulatory mechanisms of ZEP, the irreversible degradation of ZEP, in parallel with the PSII core protein D1 was observed in various plant species during very strong light conditions^77^. In these conditions, ZEP could be additionally inactivated by reactive oxygen species (ROS), which possibly irreversibly oxidize redox-sensitive cysteine residues^77^. Such a degradation or permanent ZEP inactivation, besides NADPH shortage, could explain the sustained quenching (NPQ_s_) and Dtx presence in diatoms, as observed after prolonged strong light conditions and or supra-optimal temperatures^32,36, 37,39,78^. In our conditions, however, no substantial sustained quenching was observed, as we have used the shortest possible exposure times to reach steady-state conditions and the absence of irreversible inhibition of ZEP was demonstrated by the absence of hysteresis in the NPQ values after a high light period (Fig. 1).

In this work, we only studied the epoxidation and de-epoxidation rate constants when the enzymes reached their steady-state rate constants at a given light intensity. We considered that once such a steady-state was reached, the NPQ decay became a mono-exponential curve, because of the first order kinetic reactions for the epoxidation and the de-epoxidation reactions. However, the duration of the lag before such a situation is reached can be very informative on the speed at which the XC enzymes respond to a change of light intensity. During the first minutes after high light exposure, for example, we observed a clear lag in NPQ decay (which has been removed before fitting, see Material and Methods) before a clear mono-exponential decay became evident. This lag can be explained by the time required to shift ZEP from a downregulated to a more active state, or to shift the VDE from active back to inactive. This has been reported earlier by Goss et al., and attributed due to an initial shortage of the ZEP cofactor NADPH^41^ as the Calvin-Benson-Basham cycle, functioning as an NADPH sink in high light, is not yet inactivated^41^. Also Grouneva et al. proposed a complete inhibition of ZEP in darkness after high light exposure in *P. tricornutum*^51^. Ruban et al. observed that this lag depended on the duration of the high light exposure and suggested that a “molecular lock” was involved^54^. Given the fact that we observed this lag mainly at the two highest light intensities – where the VDE is activated—we favor the explanation that the lag in the shift from high light to a lower light irradiance originates from the time required for VDE and ZEP to reach their steady-state values, i.e. 1) the time for the ΔpH generated in high light conditions to be consumed, 2) the time required for the switch of the VDE from dimeric back to monomeric form, or 3) the time for the mechanism of ZEP inhibition to reverse.

We unraveled the enzymatic rate constants of VDE and ZEP in the context of a single NPQ component ‘qZ’ and indeed verified the linearity between NPQ and Dtx. While this is evidently the major NPQ component in *P. tricornutum*, the presence of another NPQ component, similar to the qE component (exhibiting a fast relaxation and not being proportional to Dtx) in plants, has been demonstrated in other diatom species^45^. In addition, deviations from linearity between NPQ and Dtx were also reported in particular conditions in *P. tricornutum*^26,40^**.** We also observed such deviation in ML acclimated cultures, where a fast relaxation phase (< 1 min) was observed followed by a short lag before a mono-exponential decay when switching from high light conditions to darkness. Interestingly, this fast relaxation phase was also observed by Goss et al., but was not discussed^41^. This fast-relaxing NPQ component had similar characteristics as the fast-relaxing component in darkness in the centric diatom *Cyclotella meneghiniana*^45^ and is dependent on Dtx as well, as it could be inhibited with DTT and relaxed in a time frame of 10 s of seconds (data not shown). Because of this small deviation of NPQ/Dtx linearity, we decided not to use the NPQ steady-state levels and relaxation kinetics to resolve the ke and kd light-dependency in these conditions. Such a fast-relaxing component is clearly present in centric diatoms and might also exist in *P. tricornutum,* but ‘qZ’, which is characterized by a linear relationship with Dtx, is by far the main component in diatoms. Diatoms for which a linear relationship between NPQ and Dtx is observed should therefore be considered as the model species for studying this qZ mechanism and the xanthophyll cycle, which appears to be a lot more difficult in species like plants where several NPQ components are superimposed.

One of the most surprising outputs of this method is the effect of an uncoupler on the activity of the two XC enzymes. Contrary to what is usually assumed, we did not observe an inhibition of the VDE in the presence of ammonium chloride, but instead an acceleration of the ZEP. The acceleration of ZEP in the presence of ammonium chloride under high light was already demonstrated^41^, but the uncoupler effect on VDE was, for lack of means of measuring it directly, assumed rather than proven. We do not claim that this experiment rules out the possibility of a ΔpH regulation of the VDE. To check this, measuring the effect of uncouplers on the VDE rate under higher light intensities would be necessary, but is not permitted by our protocol (which is based on NPQ relaxation measurements and therefore cannot be used at light intensities generating maximal NPQ). This observation, however, shows at least that the effect of pharmacological treatments on diatom NPQ should not be analyzed as only affecting VDE regulation. We observed that the inhibition of the ZEP occurs at lower intensity than the activation of VDE; therefore at the light intensity we used here, just above the onset of NPQ, it is reasonable to propose that the generated NPQ results from the early inhibition of ZEP rather than from an activation of VDE. Moreover, in agreement with the shift to a higher pH of the pH dependency of the VDE activity measured in vitro^48^, the VDE could already be active at the neutral pH imposed by the uncoupler. The effect of the uncoupler on the activity of ZEP remains elusive. An influence of the lumenal or stromal pH on ZEP activity was proposed^41^; but an accumulation of reducing power in the form of NADPH (due to the lack of ATP for CO2 fixation) could also explain the acceleration of the ZEP. Alternatively, the influence of the uncoupler-mediated suppression of respiratory ATP could also be envisaged, in the context of the strong energetic coupling between the plastid and the mitochondrion in diatoms^55^.

In this study we used fluorimetry as a useful non-destructive tool to resolve the epoxidation and de-epoxidation enzymatic rate constants. Even though in semi-chemostat cultures considerable amounts of Dtx were present in the absence of NPQ like in polar diatoms^39^, the linear relationship between Dtx and NPQ allowed us to probe Dtx (or DES) through NPQ values. This method may be used to unravel molecular players in NPQ regulation in *P. tricornutum* and to study other photosynthetic organisms with a linear relationship between de-epoxidized xanthophylls and NPQ such as the brown alga *Macrocystis pyrifera*^79^, the alveolate *Chromera velia*^80^ or the haptophytes *Phaeocystis sp.*^81^ and *Prymnesium parvum*^81^. Alternatively, spectral reflectance^82^ could be used to probe the XC pigments when a linear relationship between de-epoxidized xanthophylls and NPQ is absent.

## Supplementary Information

Supplementary Information.

## Data Availability

The datasets generated during and/or analyzed during the current study are available at http://www.ibpc.fr/UMR7141/.
