# Supplementary material for: The fine-tuning of NPQ in diatoms relies on the regulation of both xanthophyll cycle enzymes
Source: Sci Rep. 2021 Jun 17;11:12750. doi: 10.1038/s41598-021-91483-x (PMC8211711; doi:10.1038/s41598-021-91483-x)
Supplement: Supplementary file 1 — Supplementary Information. [file 41598_2021_91483_MOESM1_ESM.docx]

The fine-tuning of NPQ in diatoms relies on the regulation of both xanthophyll cycle enzymes

**Lander Blommaert^1,2*^, Lamia Chafai^1^ & Benjamin Bailleul^1*^**

1. Laboratory of Chloroplast Biology and Light Sensing in Microalgae, UMR 7141, Centre National de la Recherche Scientifique (CNRS), Sorbonne Université, Institut de Biologie Physico-Chimique, F-75005 Paris, France
2. Current position: NIOZ Royal Netherlands Institute for Sea Research, Department of Estuarine and Delta Systems, and Utrecht University, PO Box 140, 4400 AC Yerseke, The Netherlands

*Corresponding authors : Lander Blommaert (lander.blommaert@gmail.com) and Benjamin Bailleul (bailleul@ibpc.fr)

# **Supplementary material**


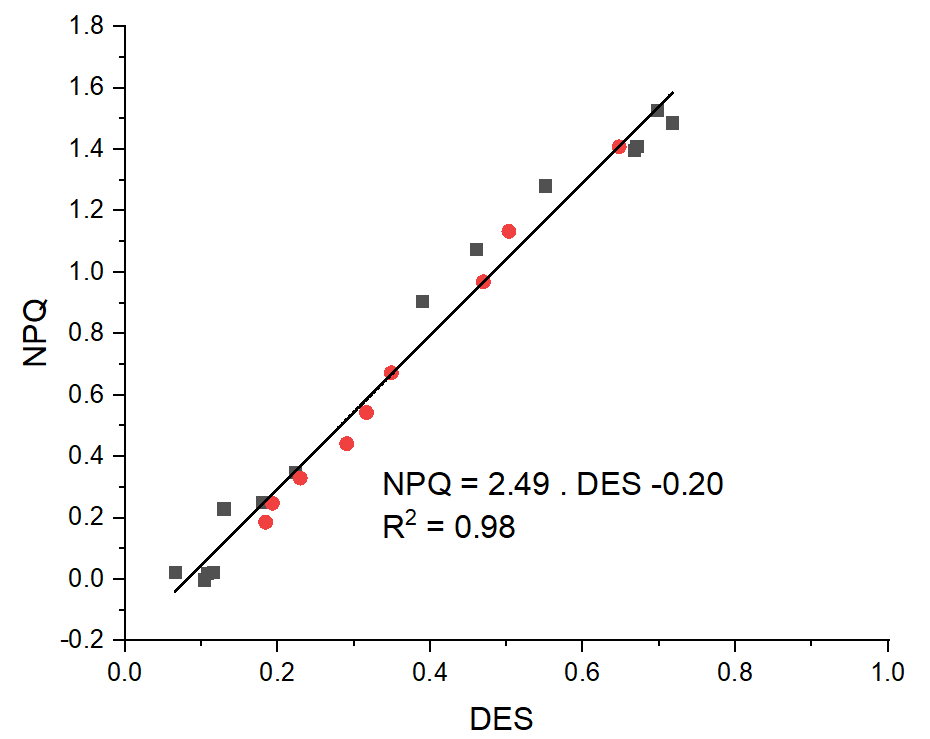


**Supplementary Figure S1:** Relationship between NPQ and concentration of Dtx, expressed as DES = [Dtx]/([Dtx]+[Ddx]), in steady-state at different light intensities (black squares) or during relaxation in darkness after an exposure to 564 µmol photons m^-2^ s^-1^ (red dots). The Dtx and Ddx concentrations were measured with HPLC (see Materials and Methods).

| a | b |
| --- | --- |
| 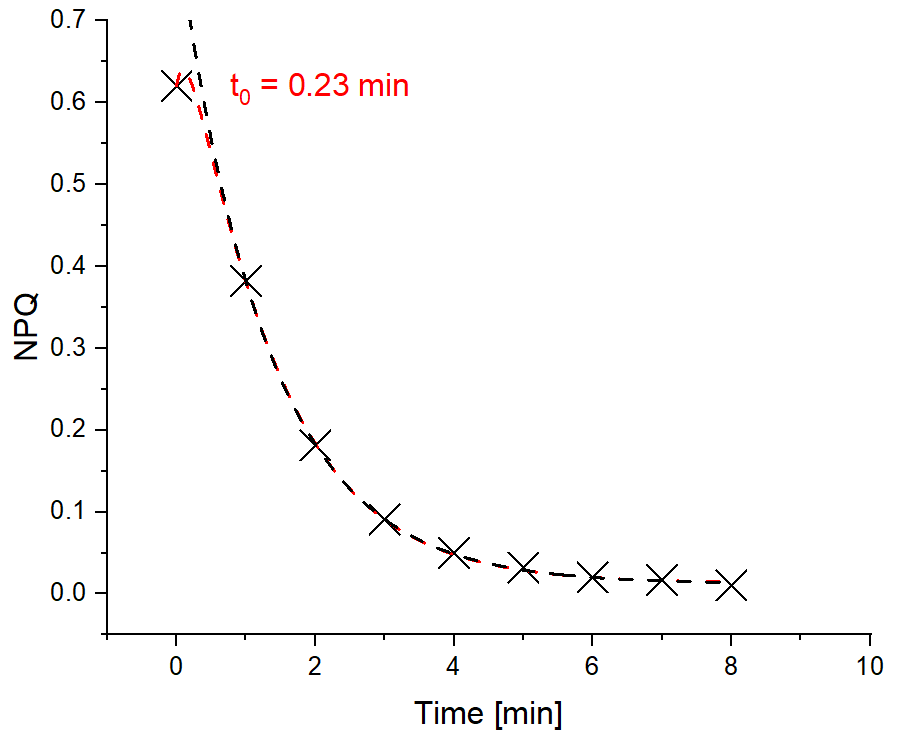 | 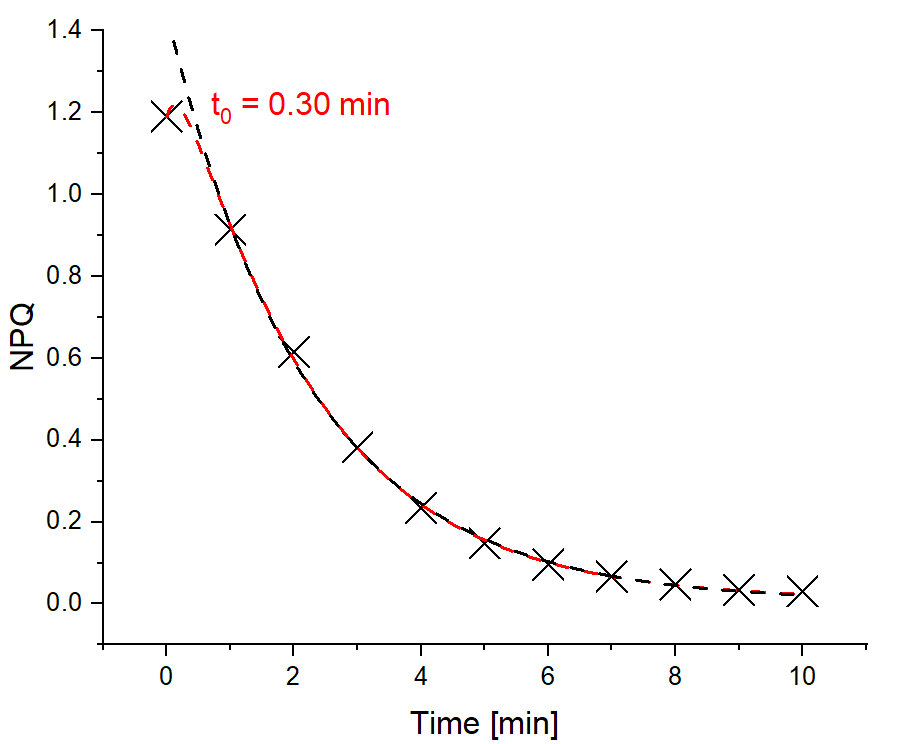 |

**Supplementary Figure S2:** NPQ relaxation kinetics of a *P. tricornutum* culture grown in semi-chemostat conditions upon the transition from 135 (a) and 231 (b) to 18 μmol photons m^-2^ s^-1^ (time zero represents the shift from preceding light to relaxation light). The dashed black curve represents the fit of a mono-exponential decay function with the first data points at (at 0 min) omitted. The dashed red curve represents our fitting function, including a lag in the exponential decay (see Material and Methods). Values of the extrapolated lag are given next to the curves.

| a | b |
| --- | --- |
| 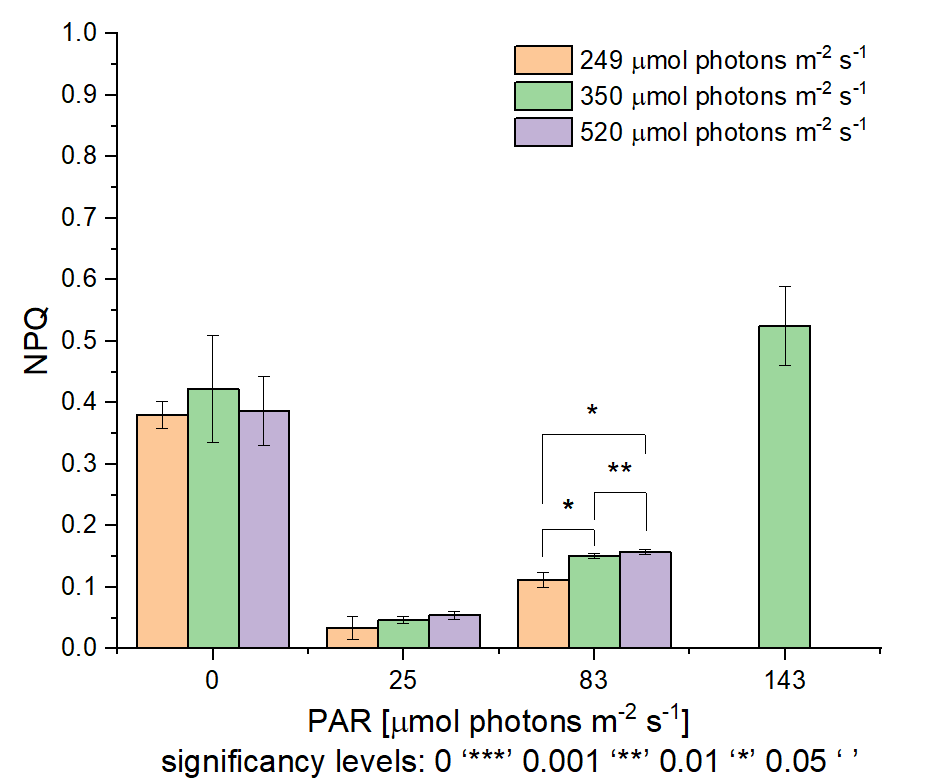 | 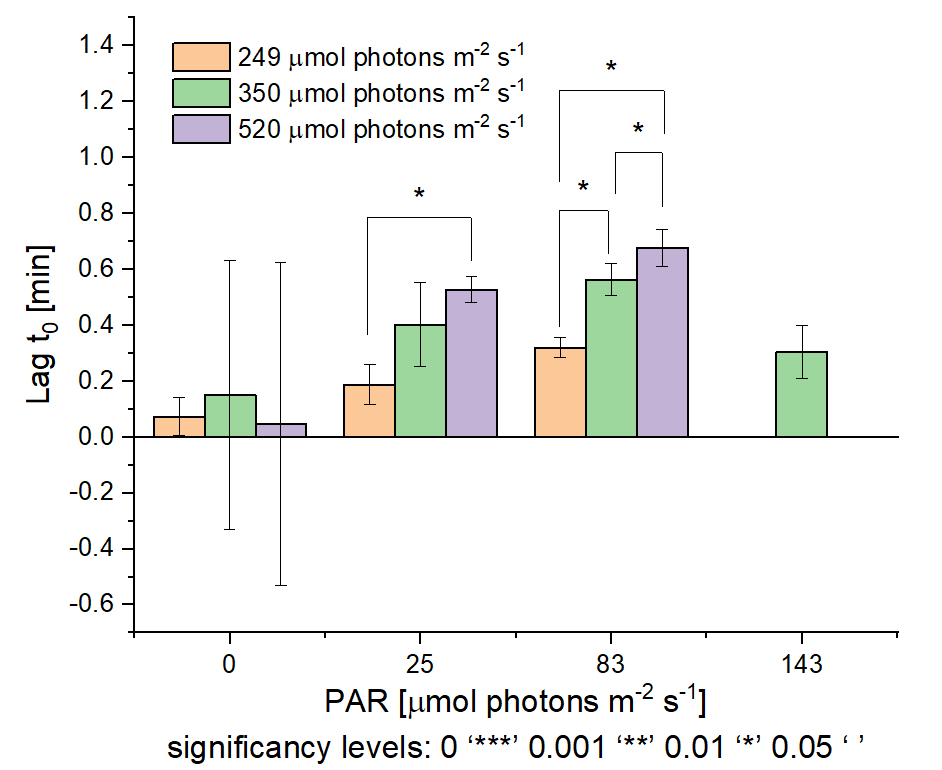 |
| c | d |
| 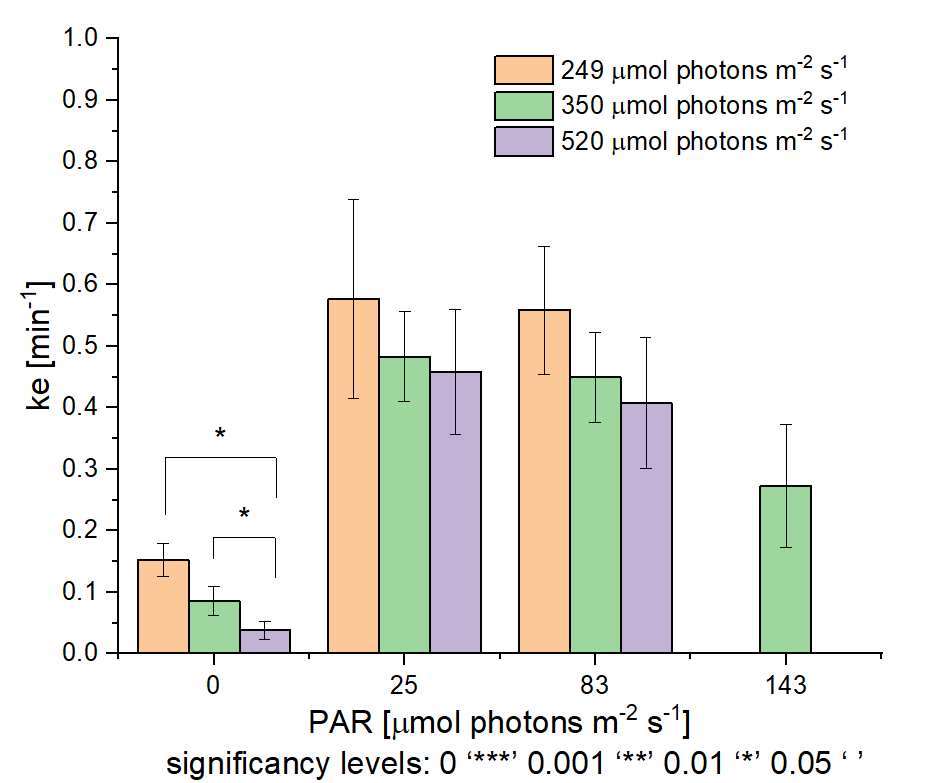 | 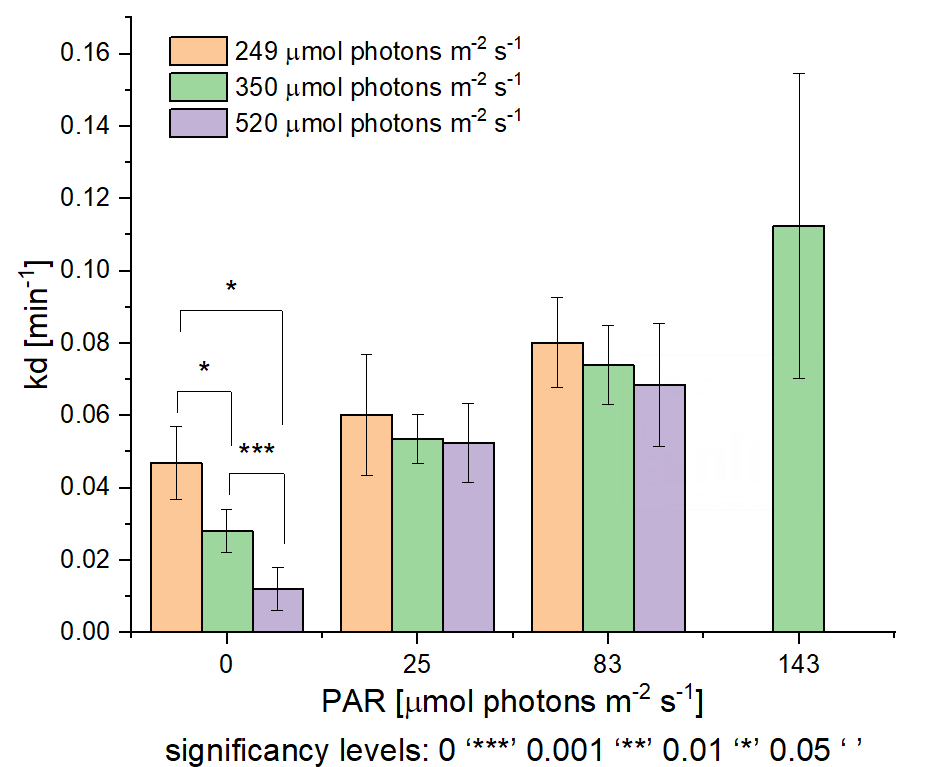 |

**Supplementary Figure S3:** Influence of the preceding light intensity on the NPQ relaxation kinetics of a *P. tricornutum* culture grown in semi-chemostat conditions. The values of the steady-state NPQ at the end of the relaxation (panel a), the lag (t0, panel b), ke (panel c) and kd (panel d) after the fitting process are shown as a function of the relaxation intensity. The different colors correspond to 3 preceding light intensities: 249 (orange), 350 (green) and 520 (purple) μmol photons m^-2^ s^-1.^. Data are mean ± SD of 3 independent biological replicates. Significant differences are indicated with * for P < 0.5; ** for P < 0.01 and *** for P < 0.001 (Paired student’s t-test).

| a | b |
| --- | --- |
| **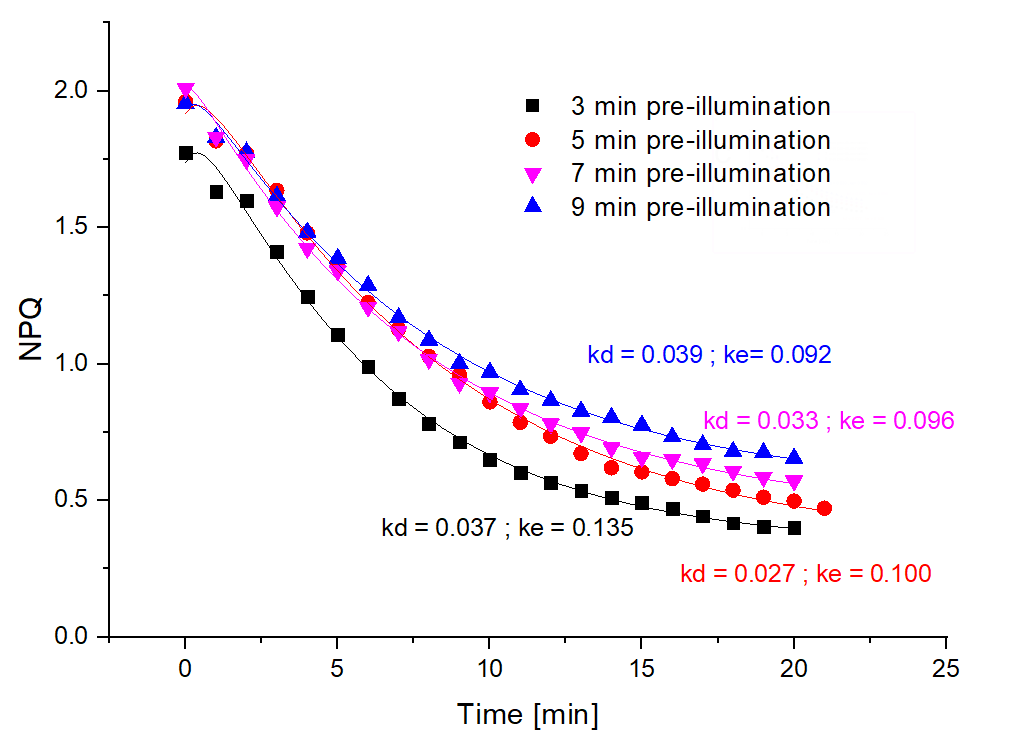** | **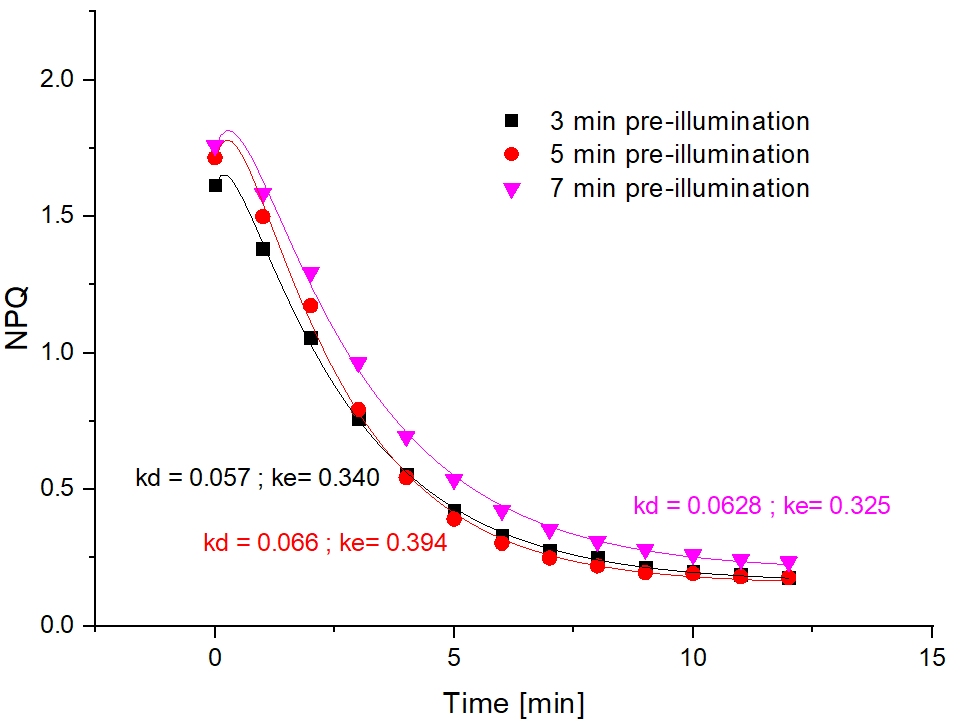** |

**Supplementary Figure S4:** Influence of the preceding light duration on the NPQ relaxation kinetics of a *P. tricornutum* culture grown in semi-chemostat conditions. Cells were pre-acclimated to a light intensity of 350 μmol photons m^-2^ s^-1^ for a duration of 3 (black symbols), 5 (red symbols), 7 (magenta symbols) or 9 (blue symbols) minutes, and then the NPQ relaxation was followed in the dark (panel a) or during 83 μmol photons m^-2^ s^-1^ (panel b) The fitting curves are shown (continuous line of the same color as the experimental datapoints) and the associated values of ke and kd are given in the same color.


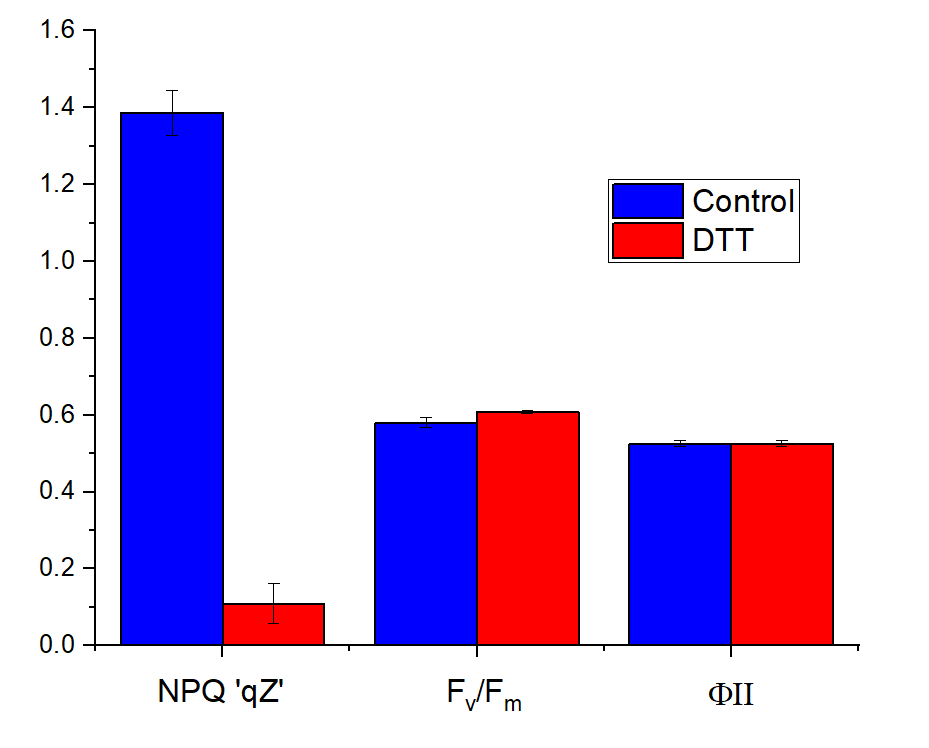


**Supplementary Figure S5:** Comparison of the reversible NPQ component qZ (see Materials and Methods), F_v_/F_m_ and ΦII (at low light of 18 µmol photons m^-2^ s^-1^) of semi-chemostat acclimated cultures in control conditions (blue bars) and in 200 μmol DTT (red bars) for cells grown in semi-chemostat mode. Values represent averages ± standard deviations of 3 independent samples.


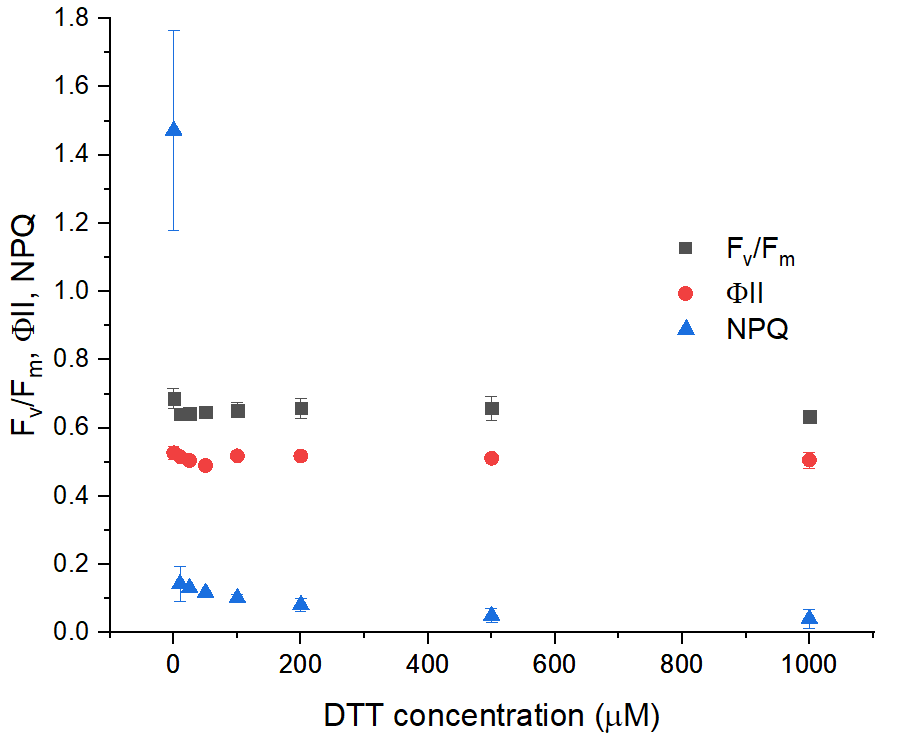


**Supplementary Figure S6:** The effect of increasing DTT concentration on NPQ (blue triangles), F_v_/F_m_ (black squares) and ΦII in 18 μmol photons m^-2^ s^-1^ (red dots) for cells grown in ML. Values represent averages ± standard deviations of 3 independent samples.

**
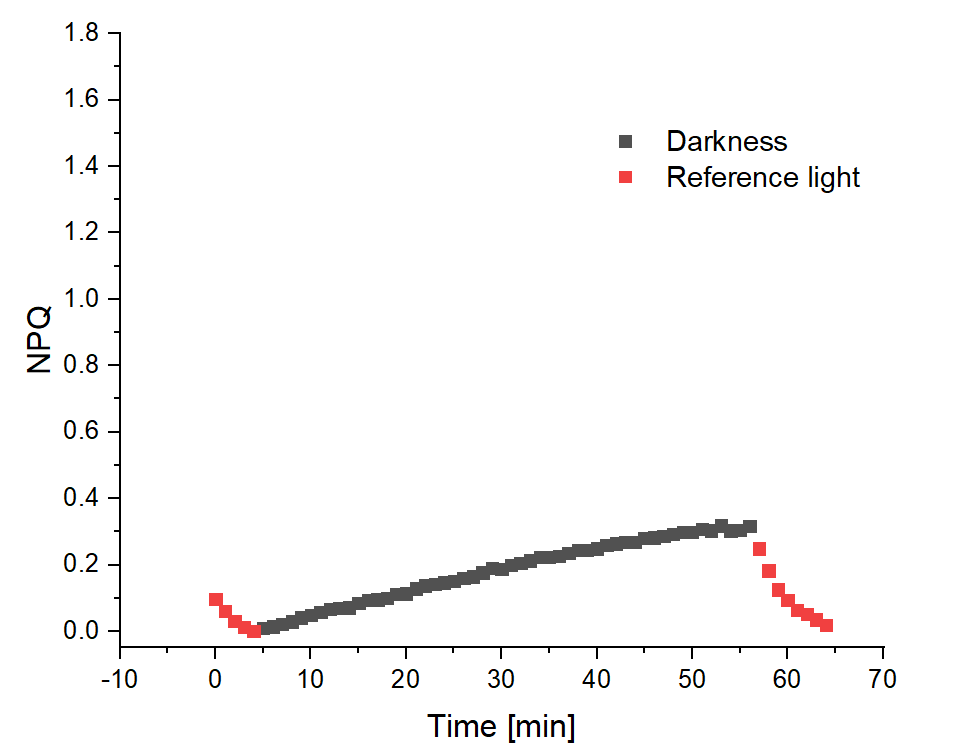
**

**Supplementary Figure S7:** The effect of prolonged dark acclimation on NPQ of a semi-chemostat culture. NPQ was relaxed in reference conditions (red symbols) before and after dark acclimation (black symbols). Only one experiment was conducted.
